# Services for people with complex psychosis: towards a new understanding

**DOI:** 10.1192/pb.bp.114.050278

**Published:** 2016-06

**Authors:** Tom Edwards, Rob Macpherson, Martin Commander, Alan Meaden, Sridevi Kalidindi

**Affiliations:** 1 Dudley and Walsall Mental Health Partnership NHS Trust; 2 2Gether NHS Foundation Trust; 3 Birmingham and Solihull Mental Health NHS Foundation Trust; 4 South London and Maudsley NHS Foundation Trust

## Abstract

This paper describes the need for commissioners and service providers to consider the development of a whole-system approach to providing rehabilitation services for patients with complex psychosis, in the context of the current economic pressures and emergence of a competitive market in this area of mental health. The practical and organisational arrangements for the management of risk with such services are described, taking into account the varying provision of rehabilitation services across the UK and considering how these can be developed against the care clustering system and interfaces with other mental health services.

Mental health rehabilitation services in the UK focus on providing locally delivered and intensive long-term care, which is cost effective and aims to maximise independence for patients with particularly complex mental health needs.^1^

A recent national survey of the provision of in-patient rehabilitation services^2^ showed that almost all National Health Service (NHS) trusts in England have at least one type of in-patient rehabilitation unit. Around 60% of these units were located in the community, 11% were wards within a psychiatric hospital and 29% were separate units within the grounds of a psychiatric hospital. The need for long-term care provision in mental health has also been acknowledged in various studies. Craig *et al*^3^ reported that around 14% of patients under an early intervention service made no recovery over the 18-month period of the study, with the implication that over time they may be likely to require longer-term care, including from rehabilitation services. Furthermore, the 10-year follow-up to the Aetiology and Ethnicity in Schizophrenia and Other Psychoses (AESOP) study also reported that 23% of 345 patients had no remission of symptoms of 6 months or more during the 10 years, and of these patients 46% had prominent negative symptoms.^4^

## Fragmentation of rehabilitation services

Across England, a range of services are commissioned for patients with severe and enduring mental illness. Such services were previously specified through the policy implementation guide produced to support functional teams,^5^ but increasingly provision is defined more by local decisions and independent of national/regional review processes. The services include:

- assertive outreach and early intervention teams
- community rehabilitation teams
- NHS rehabilitation in-patient units
- independent sector rehabilitation in-patient units
- community forensic teams.

Appropriate services, providing practical and emotional community support, housing and community-based treatments, are essential elements in the recovery-oriented care of many patients with mental health problems. In modern, post-deinstitutionalised mental healthcare, these services, which are increasingly provided through a mixed economy of NHS, private and charitable provision, are overseen by commissioning teams which are sometimes partly based in provider trusts.

However, with the current drive to reduce costs, there are pressures in many areas for specialist services to be scaled down in scope or closed. In *The Forgotten Need for Rehabilitation*, Holloway^6^ described the marginalisation of rehabilitation services. The paper appeared to foresee disinvestment from and closure of a number of English assertive outreach teams.^7^ Where this has occurred, their decommissioning teams have terminated a specific locality focus on engagement with a complex, high-risk patient group. This leaves community rehabilitation units and teams (where they exist), community forensic provision (also being cut back) and in-patient rehabilitation facilities as the only locally available support with a dedicated focus specifically for this patient group. In their absence, all patients' care defaults to community mental health teams (CMHTs), with their extensive roles and limited resources, or the wide range of charitable/private services which have arisen providing domiciliary care and accommodation support packages. Functional teams such as assertive outreach were originally developed in response to perceived failings of CMHTs, to adequately engage or reach out to marginalised and poorly treated, revolving-door patients, as in the case of Christopher Clunis.^8^ Assertive outreach teams focus on dual diagnosis and treatment-resistant psychosis, managing transitions between forensic or out-of-area placements and the long-term management of patients with impaired daily living skills and risk behaviour.

Holloway's paper^5^ also noted the diversification of rehabilitation in-patient provision involving the independent sector, often at a distance from the patient's home. In out-of-area placements a patient's rehabilitation is delivered remotely, with often limited opportunity for a more graduated rehabilitation process involving services from their home area and the ability to reintegrate patients successfully back into their local community.

Furthermore, although the closure of NHS in-patient rehabilitation units has achieved local cost savings, such decisions appear to have contributed to inappropriate use of acute in-patient psychiatric beds and often lengthy delays while alternative rehabilitation in-patient placements are identified, highlighting the likelihood that such patients have been inadequately supported in the community. Coincidentally, traditional supported accommodation models such as supported lodgings and group homes, which were part of the deinstitutionalisation movement, are now becoming outdated and this may have contributed to a perception that residential care is less ‘institutional’ when provided by non-mental health trained staff.

Such changes fragment care pathways for this patient group and it is more difficult for commissioners and NHS trust providers to monitor the care quality and cost of such services.

## Reinstitutionalisation

A study in nine European countries^9^ showed that between 2002 and 2006, the number of psychiatric beds fell but in most countries, including England, there was an increase in the number of psychiatric patients in forensic in-patient services, places in supervised/supported accommodation and in prisons. This development has been termed ‘reinstitutionalisation’, but it is important to note that the new sheltered and supported housing developments are not typically ‘institutional’ in character and less than 10% of prison inmates have psychosis.^10^ Thus, although it is possible that there may have been some reinstitutionalisation of people with mental health problems from ‘open’ hospitals to secure and high-dependency in-patient rehabilitation settings, it is unlikely that there has been much reinstitutionalisation back to prison. Increases in supervised and supported housing are not simply explained by changes in morbidity or prevalence rates of severe and enduring mental illness. Changes in family structures, which have resulted in the loss of extended support to those with mental ill health, may be a factor and concepts of mental illness may have broadened,^11^ so that people with conditions such as personality disorder may be more likely to receive support services. Alternatively, as part of a business model, private companies may have effectively widened their ‘market share’ in providing for this form of institutional care.^12^ Reinstitutionalisation indicates that whether or not commissioners pay for complex psychosis services in NHS trusts, services will need to be ensured for this patient group, whether in the public or independent sector. Despite the changing political and cultural environment, the need for services in a locality remains and appears to be relatively stable.

## New models of care

For several decades, UK government policy has increasingly promoted a market philosophy encouraging competition between different private residential care providers (not-for-profit and for-profit), at times in direct competition with state providers.^12^ This has also happened across Europe.^13^ The Supporting People strategy^14^ was set up explicitly to ring-fence and coordinate all housing support for vulnerable people, including those with mental health problems. The initiative seems to have been implemented variably, in some areas leading to greater understanding and coordination of supported accommodation, but elsewhere a lack of clear priorities has led to concerns about reduced access to services. An evaluation of the programme^15^ concluded that integrated services worked best when the service was determined by patient characteristics rather than pre-existing organisational structures, and that statutory services tended to be less flexible and more defined by professional and organisational priorities than in the voluntary sector.

A newly developed form of support for patients with severe mental illness has been the ‘tailor made’ care package. These have been developed in many areas, sometimes replacing highly supported hostel/24-hour nursed care provision. This model of supported accommodation, which has been used in some areas of the UK and also in the USA, is becoming more established in mainstream mental health services. Benefits include use of standard housing stock and a move away from transitional, staffed, shared accommodation models, which have become less acceptable to patients.^16^ Services may be provided through the voluntary sector, housing associations or private/independent organisations. Sometimes partnerships between these organisations and the NHS are in operation, with one organisation owning/running the building and another providing staffing/outreach support.

The funding and management of these placements is complex, with the involvement of both the NHS and Social Services. Some mental health providers and commissioners have specialist teams that ensure that care packages are scrutinised and move-on as well as changes are facilitated, as and when appropriate, in order to avoid stagnation of such placements.

## The forensic service interface

Violence or threatening behaviour is common in in-patient psychiatric settings and may be a precipitant for admission. A review^17^ of 11 studies found a median rate of 15% of patients committing a violent assault prior to admission (range 10–40%). It is, however, notable that a small minority of patients tend to be responsible for a large proportion of incidents.^18^ A study^19^ of problematic behaviour in a UK new long-stay population across 208 acute hospital beds identified 38 patients and of these 16% had harmed another, and 34% had been threatening or intimidating towards others.

In one study across a range of community rehabilitation units, long-term complex care units and high-dependency units,^20^ 50% of all patients had a history of serious violence or dangerousness, with this rising to 85% of the patients currently in the high-dependency units, and this was considered to be a significant barrier to discharge.

Between 1998 and 2008, the provision of mental illness beds in the NHS decreased by 62%, whereas the rate of involuntary admissions increased by 64%. Over this time, the number of non-secure beds reduced by about two-thirds, and secure bed provision increased by over 250%. The rise in involuntary admissions was due to an increase in the rate of civil involuntary admissions. However, forensic involuntary admissions fluctuated and were 15% lower at the end of this period.^21^ Provision of secure psychiatric beds in the independent sector in England has in recent times steadily increased.^22^ These changes have led to concerns regarding the costs of secure hospital care in private and third-sector hospitals, the geographical separation of these vulnerable patients from their homes, families and community backgrounds, the quality of the care provided and the availability of links to base services for monitoring and return of the patients.^23^ Attention has been drawn recently^24^ to the shift to use ‘locked rehabilitation units’ as a less expensive and less rigidly specified form of longer-term in-patient rehabilitation care.

The commissioning split between NHS England and local clinical commissioning groups has set up possible ‘perverse incentives’ which could affect patients' progress along their rehabilitation pathways. Although secure in-patient services have grown, there has been a disinvestment in community forensic services, resulting in forensic community patients receiving standard care through community mental health teams, generally with no additional training. There is now a complex and risky interface between forensic and community mental health services.

## Care clustering

As part of a move towards payment by results in mental health, the NHS directed in 2012/2013 that currencies comprising 20 care clusters would be made for the majority of adult mental health services in England. Each care cluster describes a group of patients with shared characteristics, classified with a mental health clustering tool. The cluster is linked to care packages, which have an overall cost and associated tariff. The system has been criticised, not least by the Royal College of Psychiatrists, who highlighted concerns over the lack of validity or reliability of this model of case identification, recommending specifically that diagnosis and a wider notion of complexity should also be included in the currency to improve reliability, validity and predictive value.^25^

The care clustering system is based largely on the CMHT component of the pathway. Acute in-patient, older adults and rehabilitation care pathways do not seem to have been considered and as a consequence, there is a risk of misrepresenting the care needs and subsequent funding for this particular patient group, whose needs can have associated high costs. In this context, the Royal College of Psychiatrists' Rehabilitation and Social Psychiatry Faculty, with other College specialties, is developing its own version of a clinical assessment/monitoring system. The College's position statement on clustering^25^ suggested that the process may undermine the organisation of mental health systems from a service and financial perspective. It remains unclear how far funding of mental health services will be based on the clustering system, but at this time English mental health NHS trusts are routinely collecting and reporting on these data.

The care cluster most relevant to rehabilitation in-patient units is care cluster 13 (complex needs, high support) and to community rehabilitation teams is care cluster 12 (complex needs, medium support). In addition, the care clusters most relevant for patients under the care of assertive outreach teams are care clusters 16 (dual diagnosis) and 17 (psychosis and affective disorder, difficult to engage).^26^

## The place of assertive outreach

A recent review^27^ reported on the evidence from studies outside Europe of reduced hospital bed usage, improved engagement and higher levels of satisfaction by patients under assertive outreach teams, noting that this has not been replicated by studies in the UK. The UK700 study^28^ looked at intensive case management without other aspects of assertive outreach teams and failed to deliver reductions in bed use. The REACT study^29^ included assertive outreach teams with more fidelity to the original assertive outreach model, and although reductions in bed use were noted, these did not reach statistical significance. However, it was suggested that this study was underpowered. Despite this, the REACT study also reported that assertive outreach teams might be better at engaging patients and lead to greater satisfaction with services. In a 10-year UK follow-up study of 93 patients,^27^ there was a reduction in hospital bed usage from 72 days per year prior to assertive outreach treatment to 44 days per year during assertive outreach treatment.

Other authors^30^ have suggested that the assertive outreach approach should be maintained and it appears that current provision in England is variable, with some areas moving entirely to exclusively CMHTs, some retaining full assertive outreach teams and some areas developing hybrid models such as the Functional Assertive Community Treatment Team (FACT) approach which has been deployed extensively in Holland.^31^

We believe that there remains a group of patients who, despite the national reduction of assertive outreach service provision, have a high level of need and complexity and may present challenging engagement issues and high levels of disability alongside complex risk issues. There may also be complex family pathology or other factors affecting physical health or risk. In many cases, they need a long-term intensive level of support and many would fit within the patient group described in care clusters 16 and 17.

However, with the re-integration of assertive outreach teams into CMHTs, there is a risk of these patients becoming increasingly difficult to follow up, repeating the cycle of disengagement and adverse events which led to the development of assertive outreach services in the first place. The reduced support available from a care coordinator working in a busy CMHT, coupled with the loss of the assertive outreach team approach, seems inevitably to risk disengagement and a failure to address relapse at an early stage. A team approach is crucial given the multiple needs of this patient group and is less likely to be available in already stretched CMHTs. Access to psychological interventions is particularly challenging in these services and attendance at out-patient appointments may not be feasible. There is a risk that patients with greatest levels of disability, who may be least able to express their needs, will become chronically ill and neglected in the community.

Disinvestment from assertive outreach teams in the UK has also paradoxically coincided with the development and implementation of the community treatment order (CTO) (implemented by the 2007 amendments to the Mental Health Act 1983), which in England and Wales has been taken up at a far higher rate than was expected.^32^ Although the OCTET study^33^ reported that CTOs were ineffective, others have questioned this conclusion.^34–36^ Despite this continuing wider debate, recent research^37^ has suggested that the use of the CTO in an assertive outreach context was specifically associated with a dramatic decline in hospital usage. Treatment adherence rose from 11 to 84% and the authors noted the importance of intensive case management alongside the use of the CTO.

## A whole-system approach

**Fig. 1:**
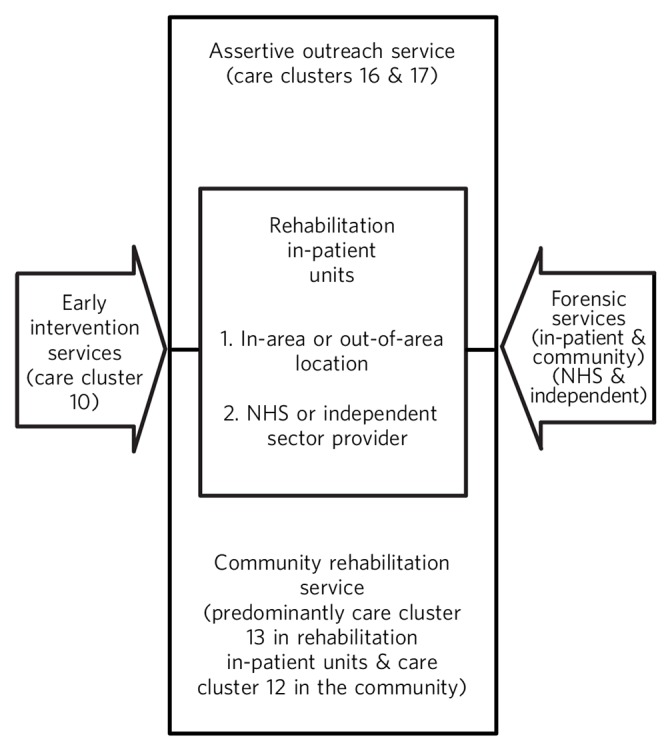
Rehabilitation services for patients with complex mental health needs. NHS, National Health Service.

The fragmentation of rehabilitation services described in this paper presents a range of challenges. Figure 1 provides a diagrammatic representation of a ‘whole system’ of support for patients who require such services. These patients could be under the care of one or more of NHS services, private companies and voluntary agencies. The relevant care clusters are configured into the diagram and show how the various parts of mental health rehabilitation services can be mapped against the care clustering system.

In practice, this is a dynamic system, with a flow of patients across the different elements, including step down from forensic services to local rehabilitation services. A whole-system approach allows the inclusion of services provided within the NHS, as well as the private and charitable sector, and implies that a failure to develop support of one type may be compensated by the provision of another. There is also an implicit suggestion that an absence of effective local services may result in an increase in the numbers of patients who require out-of-area services.

The recent separation of social care from healthcare provision in many areas of England has resulted in greater complexity in the decision-making arrangements by funding panels around costly support packages. For this whole system to work effectively, there is a requirement for cooperation across statutory and private care providers and this is a particular challenge considering the initial intention in the new commissioning arrangements to create more competition.

Rehabilitation services for patients with complex mental health needs (Fig. 1) focus on the area of rehabilitation as described in *Guidance for Commissioners of Rehabilitation Services for People with Complex Mental Health Needs*^38^ and can be adjusted within a particular geographical locality to take into account various factors, such as historical provision, local morbidity/comorbidity prevalence rates and levels of deprivation. In this context, whatever amount of contraction might have occurred in NHS rehabilitation services at a local level over time, Fig. 1 also demonstrates what provision is necessary to appropriately manage the risk and deliver the intensive levels of working required for this high-need but low-volume patient group. This can also involve patients moving to more independent living with support from the already described tailor-made care packages. Such a service would also help alleviate some of the emerging pressures within CMHTs and provide a focused commitment to the review of patients in out-of-area placements, which are coming under increasing financial scrutiny by commissioners.

The various services necessary to provide rehabilitation support for patients with psychosis and complex needs might include:

- an assertive outreach service
- a community rehabilitation service
- close interface working with forensic services
- integrated working with in-patient rehabilitation services within the NHS trust (where available)
- comprehensive out-of-area reviewing arrangements
- dedicated input into supported or semi-supported accommodation and tailor made care packages within the rehabilitation pathway.

This article is not prescriptive in the exact specifications of the operational components of such a service. This is owing to the differing levels of demand between urban *v*. rural locations, the historical reasons as to what is still available locally and also what is planned to remain in the current economic climate. However, with the demise of assertive outreach teams and the varying availability of community and in-patient rehabilitation services across NHS trusts, there is an evident need for a complex psychosis service to be developed.

## Conclusions

The absence of current government policy regarding rehabilitation services, combined with the economic pressures on the NHS and the political landscape of mixed providers operating in a progressively competitive market of rehabilitation services in the UK, has produced an increasingly complex pattern of service provision in this area of mental health. Commissioners and providers need to consider carefully which services for patients with complex mental health needs are needed and how the quality and performance of these services will be evaluated.

We strongly advocate for ‘a local, whole system, integrated rehabilitation pathway’, as described in a recent paper.^1^ The rehabilitation pathway is set within a changing health economy and needs to be dynamic and able to respond to changing patient need over time. It should ideally be combined with systems to monitor the quality and availability of services in each local healthcare region and area and to capture relevant clinical, service (including financial) and patient identified outcomes. Resource cuts create dilemmas for both commissioners and service managers and are set against the backdrop of the newly developed care clustering system. Adequate provision of services with a rehabilitation approach and a recovery orientation are needed to support these patients, with their individual paths towards recovery, over time.

## References

[1] Killaspy H The ongoing need for local services for people with complex mental health problems. Psychiatr Bull 2014; 38: 257–9. 10.1192/pb.bp.114.048470 PMC4248159 25505623

[2] Killaspy H Marston L Omar RZ Green N Harrison I Lean M The service quality and clinical outcomes: an example from mental health rehabilitation services in England. Br J Psychiatry 2013; 202: 28–34. 23060623 10.1192/bjp.bp.112.114421

[3] Craig TKJ Garety P Power P Rahaman N Colbert S Fornells-Ambrojo M The Lambeth Early Onset (LEO) Team: randomised controlled trial of the effectiveness of specialised care for early psychosis. BMJ 2004; 329: 1067–71. 15485934 10.1136/bmj.38246.594873.7C PMC526115

[4] Morgan C Lappin J Heslin M Donoghue K Lomas B Reininghaus U Reappraising the long-term course and outcome of psychotic disorders: the AESOP-10 study. Psychol Med 2014; 44: 2713–6. 25066181 10.1017/S0033291714000282 PMC4134320

[5] Department of Health Mental Health Policy Implementation Guide. Department of Health, 2001.

[6] Holloway F The Forgotten Need for Rehabilitation in Contemporary Mental Health Services. A Position Statement from the Executive Committee of the Faculty of Rehabilitation and Social Psychiatry, Royal College of Psychiatrists. Royal College of Psychiatrists, 2005.

[7] Firn M Assertive outreach: has the tide turned against the approach? Ment Health Pract 2007; 10: 24–7.

[8] Ritchie J Dick D Lingham R The Report of the Inquiry into the Care and Treatment of Christopher Clunis. HMSO, 1994.

[9] Priebe S Frottier P Gaddini A Kilian R Lauber C Martínez-Leal R Mental health care institutions in nine European countries, 2002 to 2006. Psychiatr Serv 2008; 59: 570–3. 18451020 10.1176/ps.2008.59.5.570

[10] Singleton N Meltzer H Gatward R Psychiatric Morbidity Among Prisoners in England and Wales. Department of Health, 1998.

[11] Priebe S Badesconyi A Fioritti A Hansson L Kilian R Torres-Gonzales F Reinstitutionalisation in mental health care: comparison of data on service provision from six European countries. BMJ 2005; 330: 123–6. 15567803 10.1136/bmj.38296.611215.AE PMC544427

[12] Department of Health Partnerships in Action – New Opportunities for Joint Working between Health and Social Services. A Discussion Document. HMSO, 1998.

[13] Fakhoury WKH Priebe S The process of deinstitutionalization: an international overview. Curr Opin Psychiatry 2002; 15: 187–92.

[14] Department of the Environment, Transport and the Regions Supporting People – Policy into Practice. DETR, 2001.

[15] Cameron A Macdonald G Turner W Lloyd L The challenges of joint working: lessons from the Supporting People Health Pilot evaluation. Int J Integr Care 2007; 7: 1–10. 10.5334/ijic.219 PMC2092398 18043723

[16] Rose D Muijen M Nursing doubts. Health Serv J 1997; 107: 34–5. 10168741

[17] Monahan J Mental disorder and violent behaviour: perceptions and evidence. Am Psychologist 1992; 47: 511–21. 10.1037//0003-066x.47.4.511 1595982

[18] Blumenthal S Lavender T Violence and Mental Disorder: A Critical Aid to the Assessment and Management of Risk. Jessica Kingsley Publishers, 2000.

[19] Commander M Rooprai D Survey of long-stay patients on acute psychiatric wards. Psychiatrist 2008; 32: 380–3.

[20] Cowan C Meaden A Commander M Edwards T In-patient psychiatric rehabilitation services: survey of service users in three metropolitan boroughs. Psychiatrist 2012; 36: 85–9.

[21] Keown P Weich S Bhui KS Scott J Association between provision of mental illness beds and rate of involuntary admissions in the NHS in England 1988–2008: ecological study. BMJ 2011; 343: d3736. 21729994 10.1136/bmj.d3736 PMC3130113

[22] Centre for Mental Health Pathways to Unlocking Secure Mental Health Care. CMH, 2011.

[23] Ryan T Pearsall A Hatfield B Poole R Long term care for serious mental illness outside the NHS: A study of out of area placements. J Ment Health 2006; 13: 425–9.

[24] Chukwama J Low secure versus locked rehabilitation units: a reflection. J Psychiatr Intensive Care 2015; 11: 73–7.

[25] Royal College of Psychiatrists Royal College of Psychiatrists' Statement on Mental Health Payment Systems (formerly Payment by Results) (Position Statement PS01/2014). Royal College of Psychiatrists, 2014.

[26] Kalidindi S Killaspy H Edwards T Community Psychosis Services: The Role of Community Mental Health Rehabilitation Teams (Faculty Report FR/RS/07). Royal College of Psychiatrists, 2012.

[27] Sood L Owen A A 10-year service evaluation of an assertive community treatment team: trends in hospital bed use. J Ment Health 2014; 23: 323–7. 25222169 10.3109/09638237.2014.954694

[28] UK700 Group Intensive versus standard case management for severe psychotic illness: a randomised trial. Lancet 1999; 353: 2185–9. 10392982 10.1016/s0140-6736(98)12191-8

[29] Killaspy H Bebbington P Blizard R Johnson S Nolan F Pilling F The REACT study: randomised evaluation of assertive community treatment in North London. BMJ 2006; 332: 815–9. 16543298 10.1136/bmj.38773.518322.7C PMC1432213

[30] Mortimer AM Shepherd CJ Fadahunsi A Jones A Kumar P Gangaram P Assertive outreach: mirror-image study with contemporaneous controls. Psychiatrist 2012; 36: 245–8.

[31] van Veldhuizen JR FACT: a Dutch version of ACT. Comm Ment Health J 2007; 43: 421–33. 10.1007/s10597-007-9089-4 17514502

[32] Lawton-Smith S Supervised Community Treatment (Briefing Paper 2). Mental Health Foundation, 2010.

[33] Burns T Rugkåsa J Molodynski A Dawson J Yeeles K Vazquez-Montes M Community treatment orders for patients with psychosis (OCTET): a randomised controlled trial. Lancet 2013; 381: 1627–33. 23537605 10.1016/S0140-6736(13)60107-5

[34] Curtis D OCTET does not demonstrate a lack of effectiveness for community treatment orders. Psychiatr Bull 2014; 38: 36–9. 10.1192/pb.bp.113.044800 PMC4067839 25237488

[35] Owen A Sood L Comment: OCTET does not prove community treatment orders are ineffective. Lancet Psychiatry 2015; 2: 373–5. 26360266 10.1016/S2215-0366(15)00116-9

[36] Mustafa FA The OCTET trial, community treatment orders and evidence-based practice. Psychiatr Bull 2014; 38: 197. 10.1192/pb.38.4.197 PMC4115421 25237554

[37] Rawala M Gupta S Use of community treatment orders in an inner-London assertive outreach service. Psychiatr Bull 2014; 38: 13–8. 10.1192/pb.bp.112.042184 PMC4067843 25237484

[38] Killaspy H Meier R Mitchell S Harrison C Kalidindi S Edwards T Guidance for Commissioners of Rehabilitation Services for People with Complex Mental Health Needs. Joint Commissioning Panel for Mental Health, 2012.

